# Paleosedimentary environmental reconstruction and mechanisms of the response to the Toarcian OAE in a lacustrine shale system

**DOI:** 10.1038/s41598-024-64290-3

**Published:** 2024-06-18

**Authors:** Enze Wang, Tonglou Guo, Maowen Li

**Affiliations:** 1 State Key Laboratory of Shale Oil and Gas Enrichment Mechanisms and Efficient Development, SINOPEC, Beijing, 102206 China; 2 Key Laboratory of Shale Oil/Gas Exploration and Production Technology, SINOPEC, Beijing, 102206 China; 3 Petroleum Exploration and Production Research Institute, SINOPEC, Beijing, 102206 China; 4 Southwest Oil and Gas Company, SINOPEC, Chengdu, 610041 Sichuan China

**Keywords:** Lacustrine shale oil, Biomarkers, Organic geochemical features, Sedimentary environment, T-OAE, Da’anzhai shale, Geology, Economic geology, Sedimentology, Geochemistry

## Abstract

The Lower Jurassic Ziliujing Formation in China’s Sichuan Basin is a significant shale target for exploration; however, the strong heterogeneity of the properties of organic matter (OM) in shale makes it challenging to identify the target area for exploration, and the mechanism of OM enrichment is still unclear. Furthermore, the mechanisms of the response of the Da’anzhai member to the Toarcian Oceanic Anoxic Event (T-OAE) are controversial. Previous studies have focused on sedimentary facies analysis based on mineralogy and elemental abundances and have provided minimal information about organic geochemistry, which adds to the challenge of deeply understanding the influence of the T-OAE on the molecular geochemical characteristics of the Da’anzhai member. In this study, the Da’anzhai member of the Lower Jurassic Ziliujing Formation in the Langzhong area, Sichuan Basin, is studied via X-ray diffraction, total organic carbon, gas chromatography–mass spectrometry, organic carbon isotope, organic petrographical and pyrolysis analyses. To accurately identify the trend of the paleosedimentary environmental proxies, the Mann‒Kendall test is utilized to identify the trend of the data. Our results show that the Da’anzhai shale was deposited in a dysoxic transitional environment to an intermittent reducing environment with freshwater to brackish conditions. The response to the T-OAE can be identified in the middle and upper parts of the middle submember and the bottom of the upper submember of the Da’anzhai member. The T-OAE influenced the redox conditions, salinity, and OM origins during deposition in the middle of the Da’anzhai member, which resulted in the enrichment of OM. The abnormally high C_30_ diahopane/C_30_ hopane (C_30_D/C_30_H) ratio can be considered a potential proxy for locating the section of strata that responded to the T-OAE in the Da’anzhai member. In the study area, the mechanism of the response of the Da’anzhai shale to the T-OAE manifested as an improvement in hydrological cycling rather than a marine incursion. Our study provides new information that deepens the understanding of the mechanisms of the response of lacustrine shales to oceanic anoxic events from the perspective of molecular organic geochemistry.

## Introduction

Shale oil is an unconventional petroleum resource with considerable global reserves and is regarded as an important source of energy. It is estimated that the global recoverable amount of shale oil is 618 × 10^8^ t^1^, which has attracted the attention of people in academia and industry^2–11^. Achievements in exploration provide a rationale and impetus for studying the formation mechanisms of organic-rich shales in lacustrine systems. The Da’anzhai member of the Jurassic Ziliujing Formation in the Sichuan Basin is a crucial target of lacustrine shale oil exploration^12,13^. Previous studies have confirmed that the Toarcian Oceanic Anoxic Event (T-OAE) is recorded in the Da’anzhai Member of the Sichuan Basin^14,15^. Some studies have also investigated the formation mechanisms of organic-rich shales in the Da’anzhai member^14,16,17^. However, the majority of these investigations rely on inorganic geochemical methods, with limited attention given to molecular geochemistry^15^. Compared with shales deposited in marine environments, lacustrine shales exhibit greater heterogeneity^18^. Consequently, the lack of molecular geochemical studies hinders a clear understanding of the influence of the T-OAE on the Da’anzhai member of the Sichuan Basin from the perspective of organic geochemistry.

Oceanic anoxic events (OAEs) represent significant vertical and horizontal expansions of oxygen minimum zones in most oceans in a short geological time (< 1 Myr)^14,19,20^. OAEs are typically global events; therefore, they are a vital research subject in earth system sciences. OAEs cause global disturbances in ocean-to-atmosphere systems^14^. Lacustrine sediments are sensitive to paleoclimate changes and can provide higher-resolution archives than marine sediments^18^. Therefore, lacustrine sedimentary systems provide crucial material for researching associated OAEs. Recently, lacustrine sedimentary systems associated with OAEs have attracted considerable attention from researchers^14,21–23^. Xu et al.^21^ indicated that the depositional age of the Da’anzhai member is ca. 180.3 Ma, which is consistent with the Toarcian period. Although previous studies have attempted to identify the response of Da’anzhai member to the T-OAE and determine its underlying mechanisms^14,21^, controversies about the response mechanisms still exist. Xu et al.^21^ hypothesized that marine incursions directly influenced the Da’anzhai member in the Sichuan Basin, whereas other researchers have argued that the response to OAEs in lacustrine sedimentary systems is primarily sourced from improvements in hydrological cycling^14,22,23^. Furthermore, previous studies have primarily focused on sedimentary facies analysis based on mineralogy and elemental abundance but have provided minimal information about organic geochemistry.

Differences in shale depositional environments (e.g., redox conditions, water column salinity, and organic matter sources) influence their organic matter (OM) geochemical characteristics (e.g., OM abundance and kerogen type), which determine the exploration potential of shale^24,25^. Therefore, accurate reconstruction of paleosedimentary environments is crucial for understanding OM enrichment in the shales deposited in lacustrine systems. Currently, OAE records have been identified in several lacustrine basins^14,17,26,27^. Undoubtedly, OAEs have significant impacts on OM enrichment in lacustrine shales, but the specific mechanisms involved still require further elucidation. Current methods for reconstructing paleosedimentary environments rely on organic (e.g., biomarkers) and inorganic (e.g., redox-sensitive elemental contents and ratios) geochemical methods. Biomarkers record crucial information about shales, such as OM sources and depositional environments, and are dependable representatives for the reconstruction of the paleosedimentary environment^28^. In this study, we collected borehole samples from the Langzhong area of the Sichuan Basin, conducted systematic OM mineralogical and organic geochemical analyses, and characterized the geological and geochemical features of the Da’anzhai shale. Moreover, paleosedimentary environments were reconstructed based on biomarkers, OM accumulation mechanisms were summarized, and the mechanism of the influence of the T-OAE on lacustrine systems was elucidated. The results of our study complement the molecular geochemical characteristics of the Sichuan Basin during the T-OAE period and provides new information that deepens the understanding of the mechanisms of the response of lacustrine shales to OAEs from the perspective of molecular organic geochemistry. Furthermore, our results provide a theoretical basis for the exploration of Jurassic lacustrine shale oil in the Sichuan Basin.

## Geological setting

**Figure 1:**
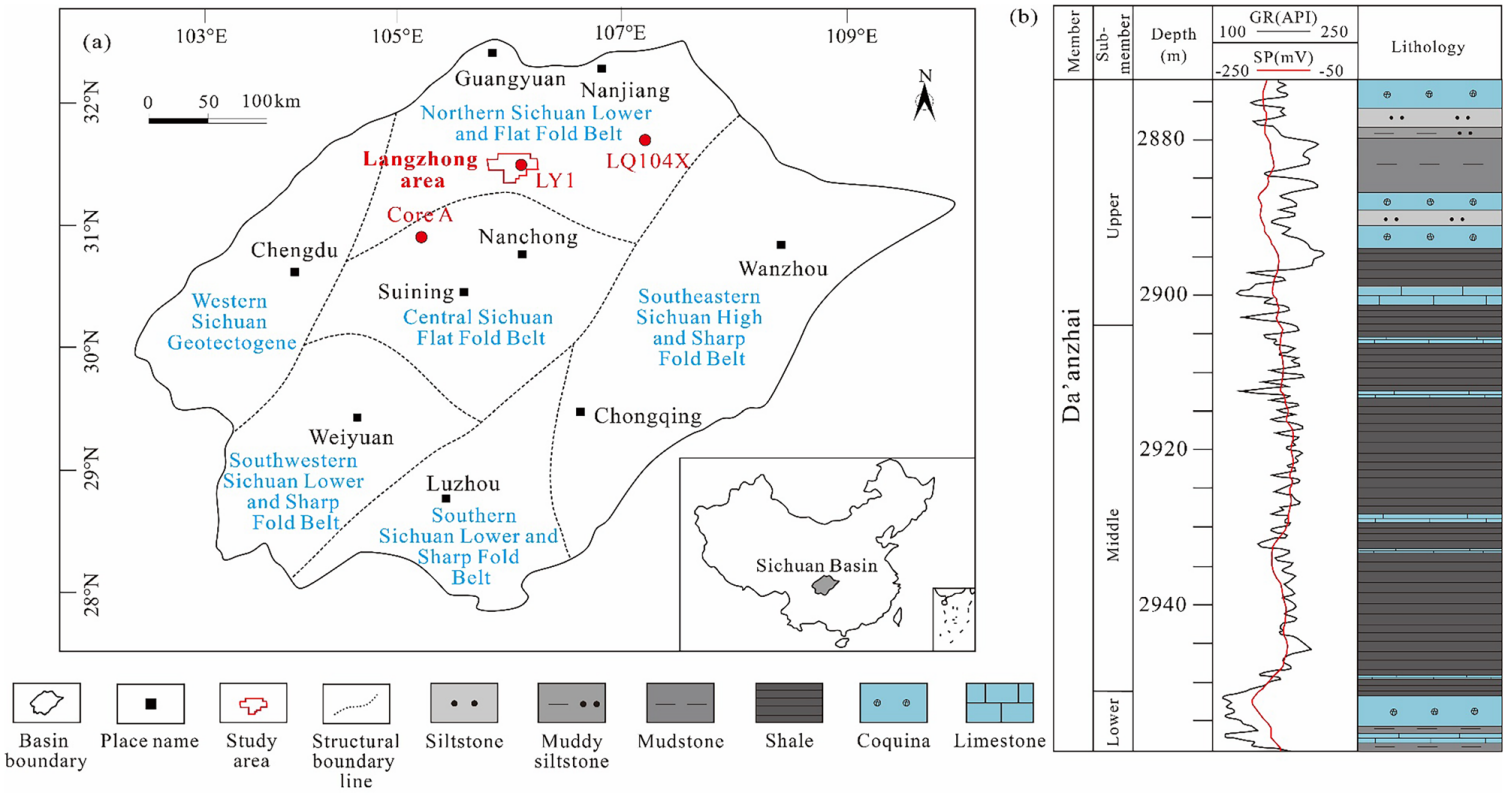
Comprehensive geological figure of the study area in the Sichuan Basin (modified from reference^7^). (**a**) Tectonic units of the Sichuan Basin and the location of the study area. (**b**) Strata column of Da’anzhai member in the study area.

The Sichuan Basin in China is petroliferous, has abundant petroleum resources and potential for shale oil exploration, and has an area of 18 × 10^4^ km^2^ (Fig. 1a)^29^. Based on the tectonic features, six substructural belts can be identified (Fig. 1a). Lacustrine deposition in the Sichuan Basin started during the Triassic^30^, and the petroleum-forming systems included the Triassic Xujiahe Formation and the Lower Jurassic Ziliujing and Lianggaoshan Formations. The Jurassic strata comprise the Ziliujing, Lianggaoshan, Shaximiao, Suining, and Penglaizhen Formations^31^. The sedimentary environment changed from a fluvial–lacustrine system to a shallow and semideep lake system from the deposition of the Triassic Xujiahe Formation to the Jurassic Ziliujing Formation^30^. The Lower Jurassic Ziliujing Formation contains four members: the Zhenzhuchong, Dongyuemiao, Ma’anshan, and Da’anzhai members. The lithology of the middle submember of the Da’anzhai member is gray–black calcareous shale interbedded with limestone, and the upper and lower submembers comprise coquina interbedded with gray mudstone, siltstone, and muddy siltstone (Fig. 1b). The study area is in the center of the Sichuan Basin and is part of the Northern Sichuan Lower and Flat Fold Belts.

## Data and methods

### Samples and experiments

Thirty-six samples were collected from the Da’anzhai member in borehole LY1 in the Langzhong area. For the middle submember of the Da’anzhai member, the majority of the samples were collected from dark gray and black shales. However, for the upper and lower submembers, the lithology of the collected samples comprised dark gray mudstone, coquina, and siltstone. The samples were evaluated using X-ray diffraction (XRD), total organic carbon (TOC), gas chromatography–mass spectrometry (GC–MS), organic carbon isotope, and pyrolysis analyses. All experiments were conducted at the SINOPEC Wuxi Institute of Petroleum Geology.

Thirty-six samples were crushed and treated with a D8 Advance X-ray diffractometer for XRD analysis, and the working voltage and current were 35 kV and 30 mA, respectively. In this study, the organic geochemistry was evaluated via TOC, pyrolysis, GC‒MS, and organic carbon isotope analyses. Thirty-six samples were crushed to 200 mesh and treated with hydrochloric acid. The inorganic carbon was removed, and the samples were analyzed using a Leco CS230 carbon/sulfur analyzer. The δ^13^C_org_ values were obtained using a Finnigan MAT 253 mass spectrometer and are reported relative to the Vienna Pee Dee Belemnite standard. After being evaluated by replicate measurements, the precision of the organic carbon isotopic analysis was better than ± 0.01‰. Organic petrographic observation was conducted using a Leica DM4500P polarizing microscope equipped with a 50 × oil immersion objective, through which the maceral compositions were identified.

A cryogenic crushing preparation technique was used to prevent the evaporation of light hydrocarbons (details about this technique can be found in Wang et al.^7^. Twenty-eight samples were selected for pyrolysis analysis using a Rock–Eval 6 pyrolysis instrument. The volatile hydrocarbon content (S_1_), pyrolyzed hydrocarbon content (S_2_), and maximum pyrolysis yield (T_max_) were obtained from the analyses.

Thirty-six samples were pretreated by Soxhlet extraction using chloroform:methanol (87:13, v/v) as the extraction solvent, and the extracted hydrocarbons were separated into saturated hydrocarbons, aromatic hydrocarbons, resin, and asphaltene. The saturated hydrocarbons were analyzed using an Agilent 6890/5973 GC‒MS using DB-5MS. The initial temperature of the GC oven was 80 °C, which was maintained for 2 min. Subsequently, the temperature was increased to 230 °C at a rate of 3 °C/min and then to 310 °C at a rate of 2 °C/min. The temperature was maintained at 310 °C for 15 min. Helium was used as the carrier gas. The ions were produced using the electron ionization (EI) technique, where the EI temperature was 230 °C, and the scan range was 50–550 (m/z) for the full scan and selective ion monitoring modes. The biomarker ratios of this study were obtained by using the peak areas in the chromatograms.

### Methods

#### Kerogen type index

For quantifying the kerogen type, the type index (TI) of kerogen obtained by organic petrographical analysis was determined in this study^32,33^, and the method of calculation is shown in Eq. (1). TI values less than 0, 0–40, 40–80, and greater than 80 represent type I, II_1_, II_2_, and III kerogen, respectively.

1 $${\text{TI}} = \frac{{S \times 100 + E \times 50 + V \times \left( { - 75} \right) + I \times \left( { - 100} \right)}}{100}$$

where S, E, V, and I are the proportions of sapropelite, exinite, vitrinite, and inertinite in the kerogen, respectively.

#### Statistical methods for quantifying the trend of data

In some studies, the identification of trends in geological data primarily depends on direct observation, which may result in trend misjudgement and data misinterpretation. To validate the presence of trends in our data, in this study, two statistical methods were employed: linear regression and the Mann‒Kendall test. Linear regression can clearly indicate whether the data trend upward or downward. For data exhibiting fluctuating features, we treated the fluctuating data as time series data and applied the Mann‒Kendall test to identify trends. This method is simple and effective because it does not require specific distributions of data and is insensitive to outliers, allowing it to accurately and quantitatively characterize the trends of the data. To demonstrate the effectiveness of the Mann‒Kendall test, this study presented two statistical parameters: the standardized normal test statistic (z value) and the p value. The z value indicates the significance of the trend, with an absolute value greater than 1.96 (or 2.58) indicating a significant trend. The p value is used to evaluate the significance of the statistical results and to determine whether the null hypothesis can be rejected. According to the Mann‒Kendall test, a p value less than 0.05 indicates the presence of a trend in the data. It is important to note that while our study still involves subjectivity in the selection of samples for trend construction, we have bolstered the scientific significance of our trends by employing statistical methods to enhance the reliability of our results.

## Results

### Petrological features and mineral composition

**Figure 2:**
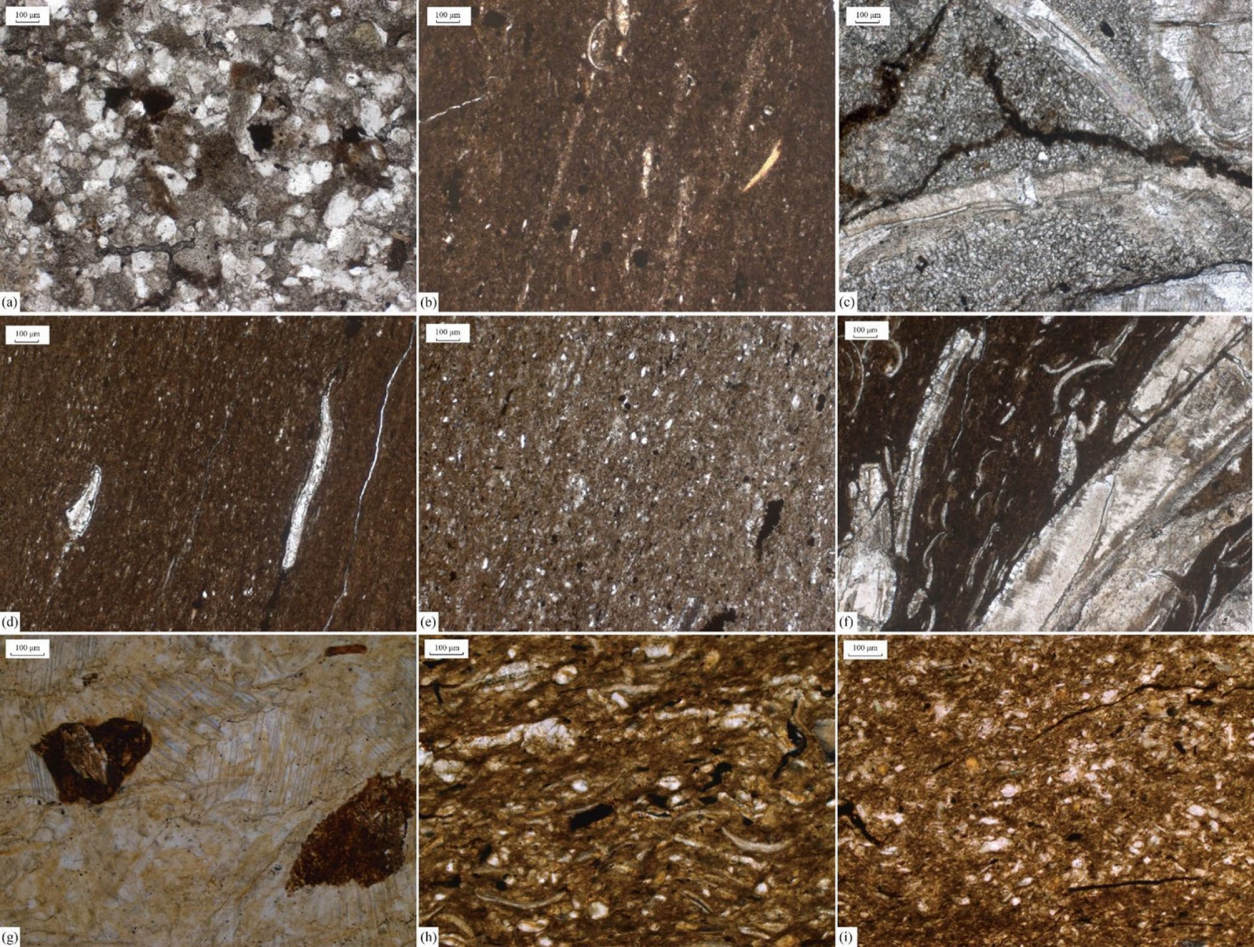
Microscopies showing the lithology of Da’anzhai shale. (**a**) upper submember, 2891.85 m, siltstone; (**b**) upper submember, 2898.23 m, shale; (**c**) upper submember, 2899.92 m, coquina; (**d**) middle submember, 2909.80 m, shale; (**e**) middle submember, 2917.54 m, calcareous shale; (**f**) upper submember, 2923.89 m, siltstone; (**g**) lower submember, 2957.24 m, siltstone; (**h**) lower submember, 2951.94 m, mudstone; (**i**) upper submember, 2957.24 m, mudstone.

Figure 2 shows the petrologic features of the Da’anzhai member. The lithology of the Da’anzhai member includes siltstone, muddy siltstone, mudstone, shale, calcareous shale, coquina, and limestone. The samples’ colors are gray to dark gray, except for the coquina, which is white to light gray. The siltstone and muddy siltstone primarily contain grains such as quartz and feldspar, with subangular shapes and good sorting (Fig. 2a). The coquina primarily contains bioclastic and calcareous minerals, but clay minerals can also be detected (Fig. 2c). The primary difference between shale and calcareous shale is the bioclastic content. In the calcareous shale, numerous oriented bioclastics can be observed (Fig. 2f), and for other shales, the bioclastic contents are significantly lower than those in the calcareous shale (Fig. 2b, d, and i). The mudstones exhibit lower OM contents and higher grain contents than the shales (Fig. 2h and i). Combined with the stratigraphic and sedimentary facies results of Liu et al.^14^, the lithological trend of the Da’anzhai member in the study area records a complete shallowing–deepening–shallowing cycle.

Table 1 shows the mineral composition. The clay minerals dominate the mineral composition of the Da’anzhai shale (the average content is 43.5%), and the middle submember has the highest average clay mineral content. The siliceous mineral contents in the Da’anzhai shale range from 3.5 to 56.6% (average of 32.3%), and the siliceous minerals are concentrated in the upper submember. The primary siliceous mineral in the sample is quartz, whereas feldspars (potassium feldspar and plagioclase) are present in lower amounts. The carbonate contents range from a trace (< 1%) to 90.0% (average 18.9%) and are highest in the lower submember. The main carbonate mineral is calcite, with minor quantities of siderite and dolomite. Aragonite was detected in only three samples, with a maximum content of 34.4%.

**Table 1:** Mineral composition of Da’anzhai shale.

Submember	Clay mineral (%)	Quartz (%)	Feldspar (%)	Calcite (%)	Dolomite (%)	Siderite (%)	Aragonite (%)	Pyrite (%)
Upper	27.6–56.8 (41.2)	28.7–55.5 (41.3)	0.8–2.3 (1.5)	0.2–33.5 (41.2)	0.2–3.2 (1.0)	0.3–1.3 (0.7)	0–3 (0.4)	0.1–3.5 (1.0)
Middle	23.5–60.7 (46.3)	10.1–41.1 (28.8)	0.6–3.4 (1.9)	0.4–50.3 (14.4)	0–12 (1.2)	0.3–2.6 (1.0)	0–36.6 (1.8)	0.6–4.9 (2.7)
Lower	6.5–40.9 (24.2)	3.5–31.5 (21.3)	0–1.6 (0.9)	23.2–89.7 (50.3)	0.3–0.7 (0.5)	0–0.7 (0.4)	0	0–2.7 (1.8)

Minimum–maximum (average value).

### Bulk geochemical and organic petrographic features

**Figure 3:**
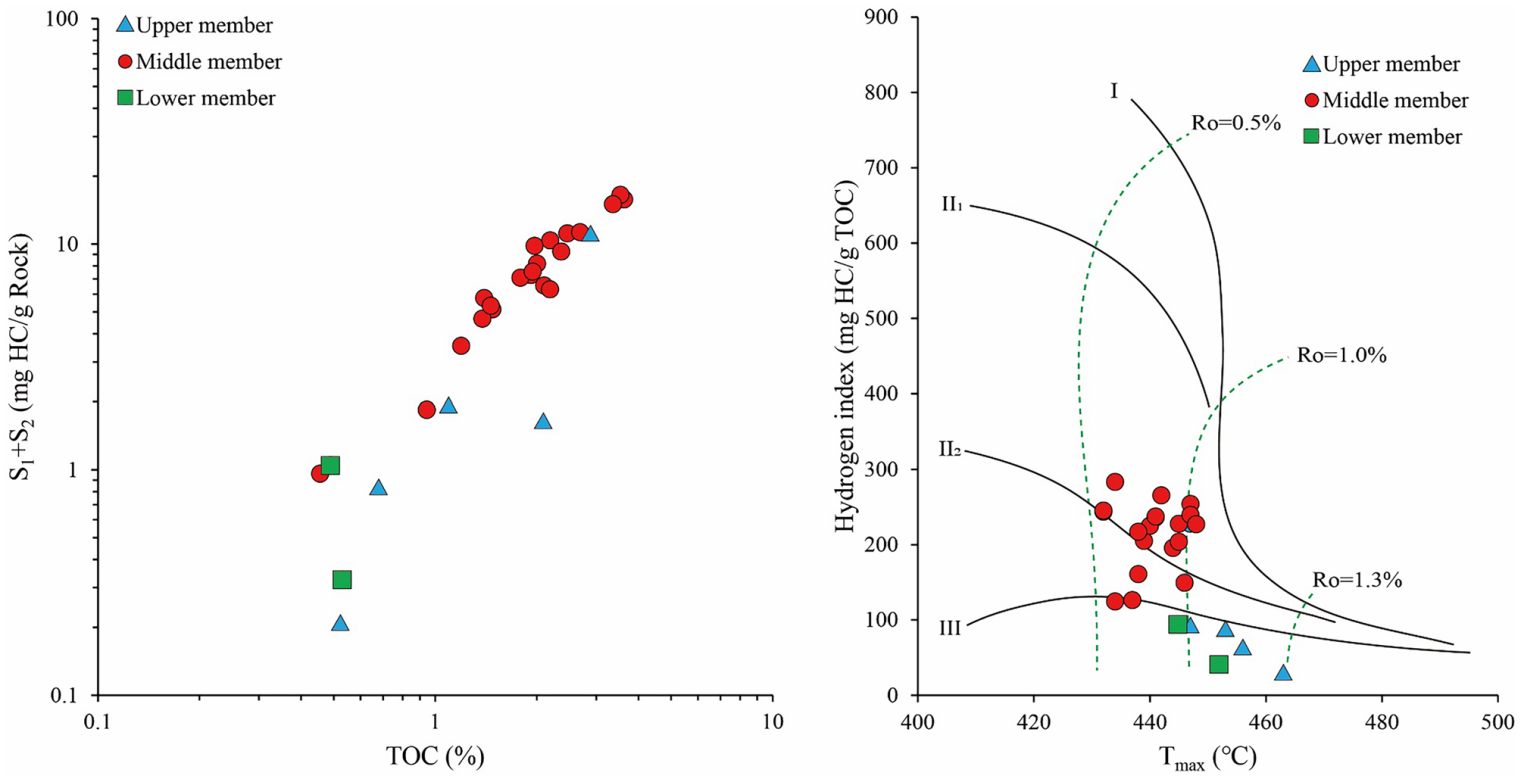
TOC content and pyrolysis parameters showing the resource quality and kerogen type of the Da’anzhai shale.

The TOC content and pyrolysis are standard indices used to evaluate the quality of shales^34,35^; our results are shown in Table 2. The TOC contents and S_1_ + S_2_ values are plotted in Fig. 3. The TOC contents of the Da’anzhai shale range from 0.10 to 3.63% (average of 1.61%). The maximum and highest mean TOC values appear in the middle submember. The S_1_ + S_2_ values vary from 0.21 to 16.53 mg HC/g rock for the Da’anzhai shale (average of 6.65 mg HC/g rock), the S_3_ values range from 0.76 to 2.91 mg HC/g rock, and the distribution patterns of S_1_ + S_2_ and S_3_ are similar to those of the TOC content. The production index (PI) values of the Da’anzhai shale range from 0.20 to 0.56, the average value is 0.41, and the S_2_/S_3_ ratios vary from 0.17 to 3.91.

**Table 2:** Bulk geochemical features of Da’anzhai shale.

Submember	Depth (m)	TOC (%)	S_1_ (mg HC/g Rock)	S_2_ (mg HC/g Rock)	S_3_ (mg HC/g Rock)	HI (mg HC/g TOC)	PI	T_max_ (°C)	δ^13^C_org_ (‰)
Upper	2874.70	0.10	–	–	–	–	–	–	− 25.47
	2880.69	0.52	0.06	0.15	0.86	27.87	0.29	463	− 23.99
	2889.67	2.10	0.32	1.29	1.14	61.63	0.20	456	− 23.07
	2890.87	1.08	–	–	–	–	–	–	− 23.18
	2896.49	0.68	0.24	0.58	1.02	85.48	0.29	453	− 26.38
	2899.07	1.09	0.90	0.99	0.88	90.74	0.48	447	− 27.41
	2902.47	2.88	4.36	6.57	2.25	227.80	0.40	447	− 29.42
Middle	2904.31	2.46	4.61	6.54	1.83	265.38	0.41	445	− 29.56
	2906.87	2.19	4.51	5.87	1.95	267.80	0.43	442	− 29.18
	2909.06	0.75	–	–	–	–	–	–	− 29.02
	2909.80	2.23	–	–	–	–	–	–	− 29.55
	2912.85	0.46	0.39	0.57	0.76	124.44	0.41	426	− 28.90
	2913.56	2.00	3.45	4.74	1.43	236.28	0.42	434	− 29.65
	2915.12	1.92	2.14	5.16	2.06	268.78	0.29	441	− 29.83
	2915.86	1.23	–	–	–	–	–	–	− 29.25
	2916.72	1.07	–	–	–	–	–	–	− 28.83
	2917.91	1.48	2.10	3.03	1.48	205.03	0.41	436	− 29.12
	2920.25	2.69	4.74	6.54	2.22	243.69	0.42	439	− 28.73
	2922.68	2.36	3.47	5.79	2.08	245.19	0.38	432	− 28.98
	2923.89	1.40	2.62	3.14	1.97	224.86	0.45	432	− 28.58
	2924.91	1.97	4.24	5.58	1.75	283.33	0.43	440	− 28.67
	2925.93	1.38	2.61	2.06	1.63	149.24	0.56	434	− 28.74
	2928.28	1.46	2.15	3.17	1.44	217.22	0.40	446	− 28.12
	2930.08	3.63	6.56	9.21	2.35	253.98	0.42	438	− 28.64
	2932.11	1.19	1.20	2.33	1.26	195.51	0.34	447	− 27.46
	2934.81	2.10	3.16	3.38	1.79	160.96	0.48	444	− 26.81
	2936.05	3.54	8.14	8.39	2.91	237.23	0.49	438	− 26.12
	2938.35	3.36	6.97	8.05	2.44	239.52	0.46	441	− 27.41
	2939.36	1.79	3.04	4.06	1.62	226.98	0.43	447	− 28.42
	2940.55	–	–	–	–	–	–	–	− 26.44
	2942.44	0.94	0.65	1.19	1.17	126.51	0.35	448	− 25.85
	2943.32	1.94	3.60	3.96	1.46	203.64	0.48	437	− 27.96
	2944.67	2.19	2.61	3.68	2.69	168.32	0.41	445	− 26.91
	2945.65	0.85	–	–	–	–	–	–	− 26.58
	2946.04	–	–	–	–	–	–	–	− 27.58
	2947.40	–	–	–	–	–	–	–	− 27.37
	2947.78	–	–	–	–	–	–	–	− 26.02
Lower	2950.53	0.14	–	–	–	–	–	–	− 28.13
	2953.48	0.49	0.59	0.46	0.79	94.00	0.56	445	− 25.97
	2958.21	0.53	0.11	0.22	1.03	40.74	0.33	452	− 24.48

*TOC* total organic carbon, *HI* hydrogen index, *PI* production index.

The hydrogen index (HI = S_2_/TOC × 100) and the T_max_ values obtained from pyrolysis are used to identify the kerogen types of the shales^36^ (Fig. 3). The HI values of the Da’anzhai shale are 27.87–283.33 mg HC/g TOC (average of 184.72 mg HC/g TOC). The T_max_ values are mainly in the range of 440–460 °C. Our results show that type II_2_ and type III kerogens dominate the Da’anzhai shale, and these shales are in the mature to high–mature stage.

**Figure 4:**
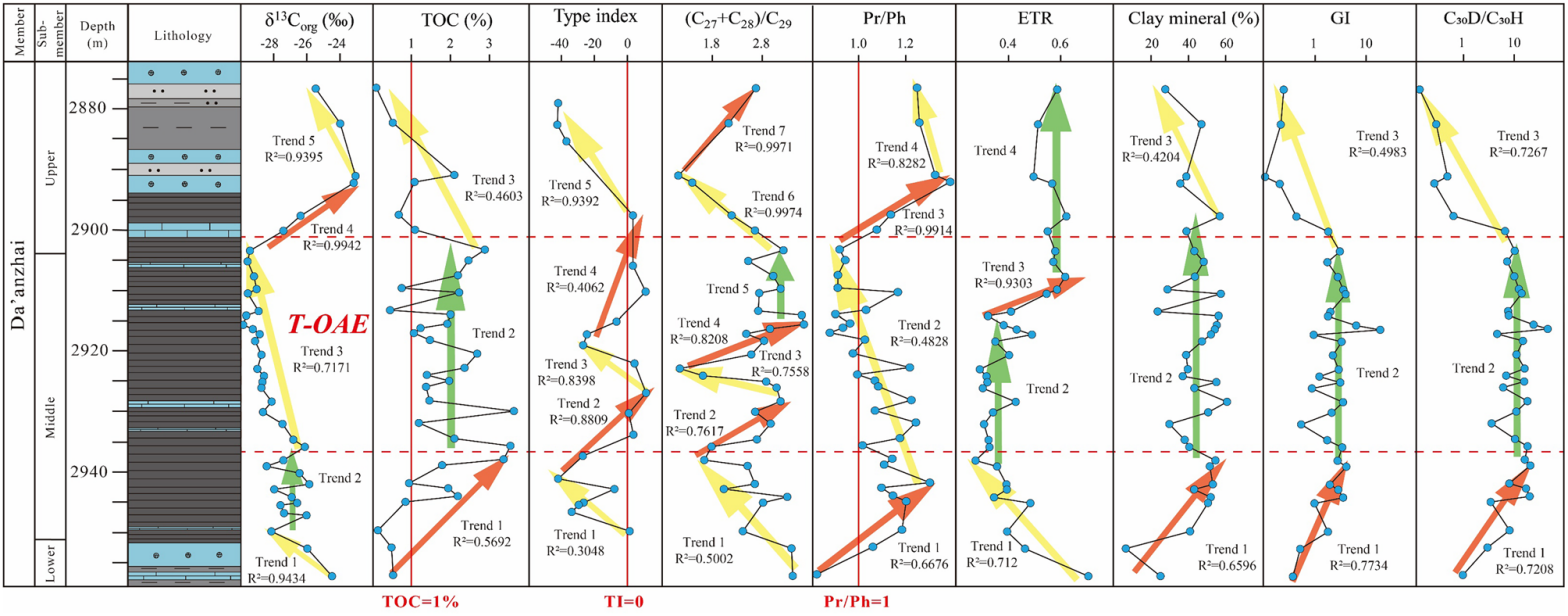
Geochemical features profiles of the Da’anzhai shale.

The δ^13^C_org_ values of the Da’anzhai shale in our study area range from − 29.8 to − 23.1‰, and our data show a negative carbon isotope excursion of 3.4‰, starting at a depth of 2936.05 m. The minimum δ^13^C_org_ value reaches − 29.8‰ at a depth of 2915.12 m and increases to − 23.1‰ at 2889.67 m. Table 2 and Fig. 4 present the detailed data and vertical distribution of the δ^13^C_org_ values.

**Figure 5:**
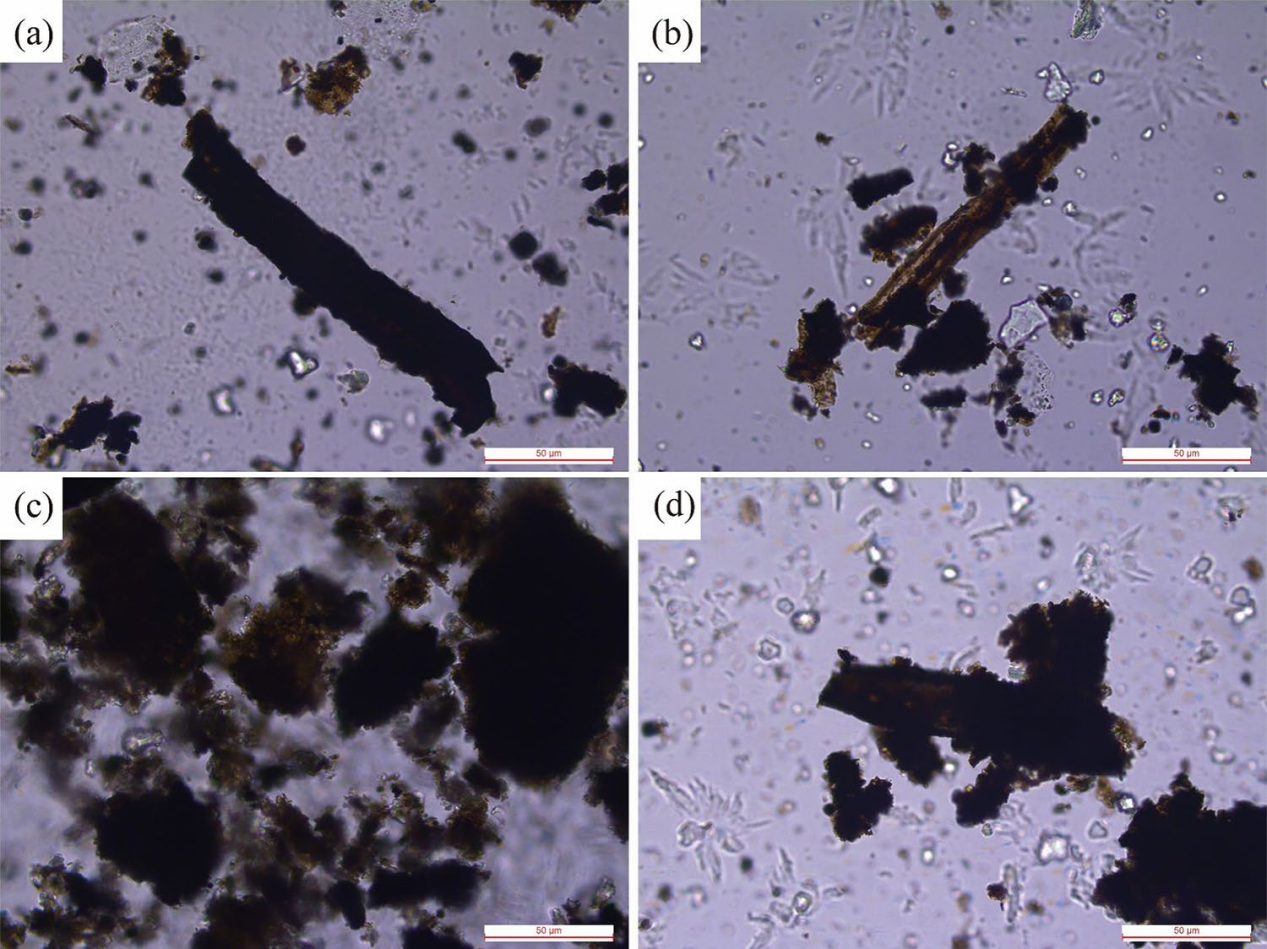
Organic petrographical analysis of Da’anzhai member in the study area. (**a**) 2883.80 m, vitrinite and humic amorphogen; (**b**) 2896.49 m, vitrinite and humic amorphogen; (**c**) 2909.55 m, Humic amorphogen; (**d**) 2941.55 m, vitrinite and humic amorphogen.

The organic petrographic analyses of the Da’anzhai shale are shown in Table 3 and Fig. 5, and there is almost no sapropelite or inertinite in the samples. The exinite shows structureless characteristics (Fig. 5c). The vitrinite includes structural and structureless vitrinite (Fig. 5a, b, d). The main differences in the dataset in this study are the proportions of exinite and vitrinite, and the TI values of the Da’anzhai shale range from − 42.5 to 11.5.

**Table 3:** Organic petrographical analysis result of Da’anzhai shale.

Submember	Depth (m)	Sapropelite (%)	Exinite (%)	Vitrinite (%)	Inertinite (%)	Type index
Upper	2877.26	0	28	64	8	− 42.0
	2880.96	0	28	62	10	− 42.5
	2883.80	0	32	60	8	− 37.0
	2896.49	0	64	30	6	3.5
Middle	2905.12	0	64	30	6	3.5
	2909.55	0	70	25	5	11.3
	2914.63	0	53	38	6	− 6.5
	2916.82	0	42	50	8	− 24.5
	2918.66	0	40	53	7	− 26.8
	2921.80	0	65	28	7	4.5
	2926.84	0	70	26	4	11.5
	2930.31	0	62	32	6	1.0
	2933.99	0	64	31	5	3.8
	2937.65	0	40	52	8	− 27.0
	2941.55	0	28	64	8	− 42.0
	2943.32	0	55	39	6	− 7.8
	2945.65	0	40	54	6	− 26.5
	2946.04	0	38	54	8	− 29.5
	2947.24	0	35	55	10	− 33.8
Lower	2950.53	0	62	34	4	1.5

### Molecular geochemistry analysis

**Figure 6:**
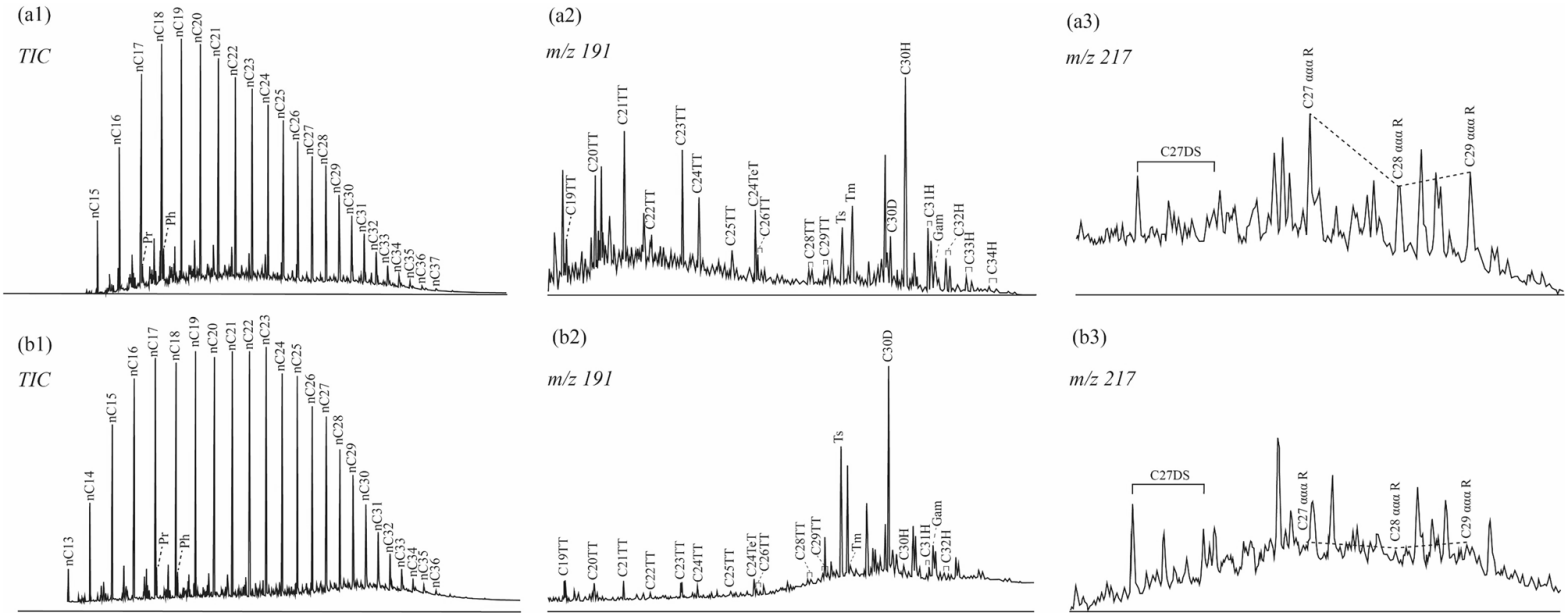
The total ion chromatograms and representative chromatograms of saturated hydrocarbons of Da’anzhai shale. (**a**) 2880.69 m, Da’anzhai member, TOC = 0.52%; (**b**) 2922.68 m, Da’anzhai member, TOC = 2.36%.

Figure 6 shows the total ion and representative chromatograms of the saturated hydrocarbons of the Da’anzhai shale. Table 4 shows some biomarker features. Normal alkanes are in the n-C_13_–C_37_ range. Some samples show unimodal distributions with low–moderate-molecular-weight n-alkane peaks (such as the n-C_16_ and n-C_19_ peaks, Fig. 6a1), whereas other samples exhibit bimodal distributions (Fig. 6 (b1)). The first peak corresponds to low-molecular-weight (< n-C_20_) n-alkanes (n-C_19_), and the second peak corresponds to moderate-molecular-weight (n-C_21_–n-C_25_) n-alkanes (n-C_23_). The abundance of the front peak is greater than that of the trailing peak. The (nC_21_ + nC_22_)/(nC_28_ + nC_29_) ratios range from 0.52 to 3.07, and the terrigenous/aquatic ratios (TAR = (nC_27_ + nC_29_ + nC_31_)/(nC_15_ + nC_17_ + nC_19_))^37^ range from 0.25 to 3.21. The samples’ carbon preference index (CPI) and odd-to-even predominance (OEP) values are close to 1, showing no significant differences among the different submembers of the Da’anzhai member samples. The pristane/phytane (Pr/Ph) ratios are 0.82–1.39.

**Table 4:** Normal alkane, isoprenoids, and related parameters of Da’anzhai shale.

Submember	Depth (m)	TOC (%)	CPI	OEP	Main peak carbon	Pr/Ph
Upper	2874.70	0.10	1.07	0.94	nC23	1.25
	2880.69	0.52	1.08	1.00	nC19	1.26
	2889.67	2.10	1.06	0.97	nC19	1.33
	2890.87	1.08	1.11	1.28	nC14	1.39
	2896.49	0.68	1.11	1.00	nC17	1.14
	2899.07	1.09	1.11	0.99	nC20	1.08
	2902.47	2.88	1.07	0.99	nC23	0.92
Middle	2904.31	2.46	1.08	0.98	nC23	0.94
	2906.87	2.19	1.08	0.98	nC23	0.91
	2909.06	0.75	1.08	0.99	nC23	0.91
	2909.80	2.23	1.08	0.97	nC21	1.17
	2912.85	0.46	1.10	0.99	nC23	1.03
	2913.56	2.00	1.09	0.98	nC22	0.90
	2915.12	1.92	1.09	1.00	nC23	0.96
	2915.86	1.23	1.07	0.99	nC21	0.93
	2916.72	1.07	1.08	1.00	nC23	0.88
	2917.91	1.48	1.10	0.98	nC21	1.03
	2920.25	2.69	1.10	1.01	nC23	0.98
	2922.68	2.36	1.08	0.99	nC23	1.22
	2923.89	1.40	1.10	0.98	nC25	0.99
	2924.91	1.97	1.09	0.99	nC23	1.07
	2925.93	1.38	1.11	0.96	nC20	1.08
	2928.28	1.46	0.95	0.99	nC17	1.22
	2930.08	3.63	1.09	0.99	nC23	1.07
	2932.11	1.19	1.10	0.99	nC23	1.24
	2934.81	2.10	1.08	0.93	nC17	1.18
	2936.05	3.54	1.08	1.01	nC23	1.02
	2938.35	3.36	1.11	1.01	nC23	1.14
	2939.36	1.79	1.06	0.99	nC23	1.11
	2942.44	0.94	1.10	1.02	nC17	1.30
	2943.32	1.94	1.05	1.00	nC23	1.10
	2944.67	2.19	1.07	0.99	nC21	1.15
	2945.65	0.85	1.10	0.99	nC20	1.20
Lower	2950.53	0.14	1.12	1.01	nC23	1.18
	2953.48	0.49	1.08	1.27	nC16	1.06
	2958.21	0.53	1.11	0.99	nC19	0.82

*CPI* carbon preference index, *OEP* odd-to-even predominance, *Pr/Ph* Pristane/phytane.

Common tricyclic terpanes (TTs, *m/z* 191), namely, C_19_TT–C_26_TT, are present in the extracted hydrocarbons. Moreover, high-carbon-number TTs (C_28_TT and C_29_TT, *m/z* 191) and C_24_ tetracyclic terpanes (TeT, *m/z* 191) are present (Fig. 6a2). The abundance of TTs varies considerably (Fig. 6a2 and b2). In most samples, the abundance of 18α(H)-22,29,30-C_27_ trisnorneohopane (Ts, *m/z* 191) is greater than that of 17α(H)-22,29,30-C_27_ trisnorneohopane (Tm, *m/z* 191), and the Ts/(Ts + Tm) ratios range from 0.31 to 0.88. C_30_ diahopanes (C_30_D, *m/z* 191) are detected in all the samples (Fig. 6a2), and the C_30_D/C_30_ hopane (C_30_H, *m/z* 191) ratios are 0.12–47.89. A high C_30_D/C_30_H ratio of the Da’anzhai member was also reported by Lu et al.^38^. Furthermore, C_31_ to C_34_ hopanes and gammacerane (Gam, *m/z* 191) are detected, and the Gam index (GI = Gam/C_30_H) values range from 0.10 to 19.11 (Table 5).

C_27_, C_28_, and C_29_ regular steranes and rearranged C_27_ steranes are present in all Da’anzhai member samples (*m/z* 217, Fig. 6 (a3) and (b3)). The relative proportions of C_27_, C_28_, and C_29_ regular steranes differ significantly among the various samples, and the (C_27_ + C_28_)/C_29_ ratios range from 1.12 to 3.63. The R and S isomers were evaluated in the C_27_–C_29_ steranes, and the C_29_ ααα 20S/(20S + 20R) and C_29_ αββ/(αββ + ααα) ratios range from 0.45 to 0.78 and 0.40–0.54, respectively (Table 5).

**Table 5:** Biomarkers of saturated hydrocarbon and aromatic hydrocarbon of Da’anzhai shales.

Submember	Depth (m)	TOC (%)	ETR	Ts/(Ts + Tm)	C_30_D/C_30_H	Gam/C_30_H	C_29_ αββ/(αββ + ααα)	C_29_ ααα 20S/(20S + 20R)	(C_27_ + C_28_)/C_29_
Upper	2874.70	0.10	0.59	0.47	0.12	0.24	0.40	0.45	2.67
	2880.69	0.52	0.51	0.42	0.26	0.21	0.44	0.50	2.13
	2889.67	2.10	0.50	0.31	0.44	0.10	0.47	0.53	1.12
	2890.87	1.08	0.57	0.40	0.24	0.20	0.46	0.51	1.40
	2896.49	0.68	0.62	0.52	0.58	0.43	0.45	0.48	2.19
	2899.07	1.09	0.55	0.72	6.53	1.82	0.48	0.68	2.65
	2902.47	2.88	0.58	0.74	10.25	3.08	0.49	0.72	3.22
Middle	2904.31	2.46	0.57	0.69	7.22	1.75	0.48	0.66	2.52
	2906.87	2.19	0.62	0.70	10.02	2.80	0.53	0.73	3.02
	2909.06	0.75	0.59	0.71	12.42	3.59	0.52	0.67	3.17
	2909.80	2.23	0.55	0.73	14.18	3.99	0.48	0.62	2.74
	2912.85	0.46	0.41	0.78	7.51	2.00	0.43	0.65	2.72
	2913.56	2.00	0.32	0.80	7.73	1.80	0.49	0.75	3.59
	2915.12	1.92	0.38	0.79	24.79	6.44	0.51	0.72	3.63
	2915.86	1.23	0.43	0.79	47.89	19.11	0.42	0.71	2.96
	2916.72	1.07	0.49	0.72	4.52	0.94	0.50	0.63	2.48
	2917.91	1.48	0.35	0.84	15.02	3.32	0.48	0.69	2.84
	2920.25	2.69	0.40	0.80	11.15	2.24	0.51	0.67	2.58
	2922.68	2.36	0.29	0.85	16.25	2.91	0.44	0.69	1.15
	2923.89	1.40	0.32	0.79	6.87	1.21	0.54	0.57	1.61
	2924.91	1.97	0.32	0.86	15.96	3.14	0.47	0.73	2.88
	2925.93	1.38	0.30	0.82	5.97	0.87	0.49	0.73	3.09
	2928.28	1.46	0.43	0.81	18.65	3.56	0.50	0.68	3.17
	2930.08	3.63	0.34	0.84	11.03	2.12	0.50	0.67	2.66
	2932.11	1.19	0.31	0.78	3.47	0.53	0.47	0.78	2.97
	2934.81	2.10	0.32	0.85	10.41	1.73	0.46	0.66	2.69
	2936.05	3.54	0.33	0.88	18.69	3.42	0.45	0.65	1.79
	2938.35	3.36	0.27	0.86	16.36	2.78	0.42	0.66	1.64
	2939.36	1.79	0.36	0.87	21.21	4.12	0.46	0.71	2.51
	2942.44	0.94	0.39	0.77	7.98	1.95	0.44	0.64	2.65
	2943.32	1.94	0.39	0.84	17.39	2.85	0.44	0.66	2.03
	2944.67	2.19	0.34	0.85	20.61	3.58	0.48	0.70	3.30
	2945.65	0.85	0.48	0.71	3.33	0.97	0.43	0.55	2.82
Lower	2950.53	0.14	0.39	0.80	8.06	1.79	0.47	0.59	2.41
	2953.48	0.49	0.46	0.72	2.87	0.51	0.46	0.58	3.39
	2958.21	0.53	0.71	0.56	0.91	0.37	0.43	0.50	3.41

ETR: Extended tricyclic terpane ratio; Ts: 18α(H)-22,29,30-C_27_ trisnorneohopane; Tm: 17α(H)-22,29,30-C_27_ trisnorneohopane; C_30_D: C_30_ diahopanes; C_30_H: C_30_ hopane; Gam: Gammacerane; (C_27_ + C_28_)/C_29_: (C_27_ regular sterane + C_28_ regular sterane)/C_29_ regular sterane.

## Discussion

### Thermal maturity

Thermal maturity considerably influences the effectiveness of biomarkers as parameters for the depositional setting; hence, it is essential to characterize thermal maturity first. The T_max_ values of the Da’anzhai shale in the study area are mainly in the range of 440–460 °C, and the variation in the T_max_ value may be attributed to differences in the hydrocarbon content rather than variations in thermal maturity. The PI ratios are mainly in the range of 0.3–0.5. Moreover, previous studies have suggested that the vitrinite reflectance (Ro) values are mainly in the range of 1.0–1.2%^33,39^. Earlier Ro results and the T_max_ values and PI ratios of our samples indicate that the Da’anzhai shales are in the mature to highly mature stage. The CPI and OEP values in our study area are approximately equal to 1, showing no odd–even predominance, indicating a mature stage of the Da’anzhai shale in the investigated area.

Furthermore, the high content of low-molecular-weight n-alkanes in most samples indicates that maturity influences biomarkers. The C_29_ αββ/(αββ + ααα) and C_29_ ααα 20S/(20S + 20R) data for the Da’anzhai shale in the study area indicate that all the samples have reached the mature stage; the high Ts/(Ts + Tm) values (mean value of 0.73) also indicate that they are the same.

### Record of the T-OAE in the Da’anzhai member

#### Trend analysis of the data

Figure 4 and Table 6 summarize the trends of the data and corresponding statistical parameters. Linear regression was used to identify clear upward (indicated by red arrows) or downward (indicated by yellow arrows) trends, with correlation coefficient (R^2^) values calculated. For fluctuating data (indicated by the green arrows in Fig. 4), the R^2^ values of the linear regression are too low to reflect any trend (Table 6). In these cases, the Mann‒Kendall test is used to determine the z value and p value of the data. All the absolute z values are less than 1.96, and all the p values are greater than 0.05, indicating that there are no clear trends and that the data exhibit fluctuating characteristics. The above statistical data indicate that the trends of the paleosedimentary environmental proxies in our study are reliable.

**Table 6:** Statistical parameters of data trend.

Parameters	Trend	Linear regression R^2^	Mann–Kendall test	
			Z-value	*P* value
δ^13^C_org_	1	0.9434	–	–
	2	0.0282	–0.52	0.602
	3	0.7171	–	–
	4	0.9942	–	–
	5	0.9395	–	–
TOC	1	0.5692	–	–
	2	0.0766	–0.487	0.627
	3	0.4603	–	–
TI	1	0.3048	–	–
	2	0.8809	–	–
	3	0.8398	–	–
	4	0.4062	–	–
	5	0.9392	–	–
(C_27_ + C_28_)/C_29_	1	0.5002	–	–
	2	0.7617	–	–
	3	0.7558	–	–
	4	0.8208	–	–
	5	0.0563	–0.751	0.452
	6	0.9974	–	–
	7	0.9971	–	–
Pr/Ph	1	0.6676	–	–
	2	0.4828	–	–
	3	0.9914	–	–
	4	0.8282	–	–
ETR	1	0.712	–	–
	2	0.2596	1.314	0.1889
	3	0.9303	–	–
	4	0.1614	–1.608	0.108
Clay mineral content	1	0.6596	–	–
	2	0.0019	–0.05	0.96
	3	0.4204	–	–
GI	1	0.7734	–	–
	2	0.0273	0.338	0.735
	3	0.4983	–	–
C_30_H/C_30_D	1	0.7208	–	–
	2	0.0138	–1.072	0.284
	3	0.7267	–	–

*TI* type index, *GI* Gam index.

#### Response of the Da’anzhai member to the T-OAE

**Figure 7:**
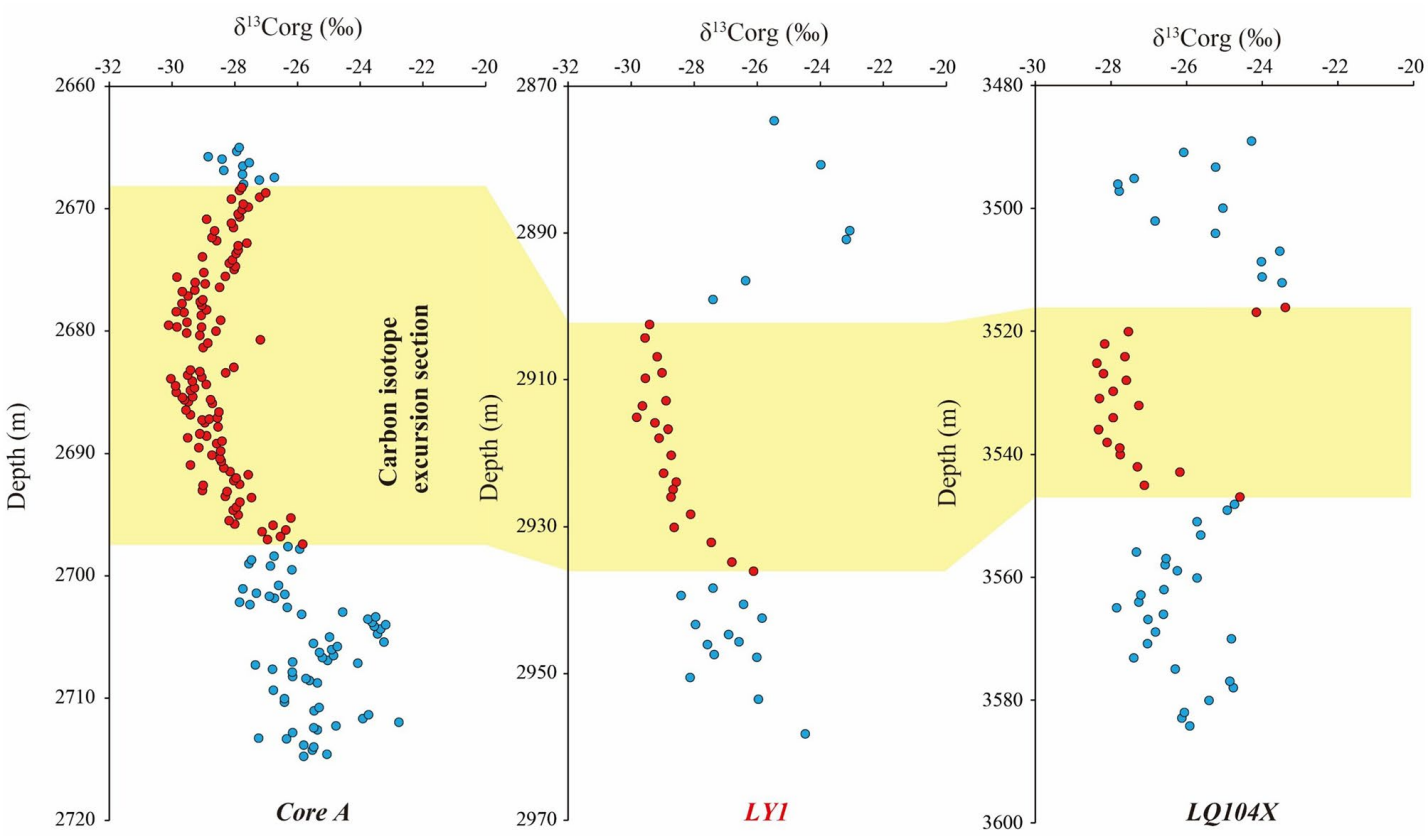
Profile shows the carbon isotope excursions of Da’anzhai member in three boreholes of the Sichuan Basin (the location of the boreholes can be found in Fig. 1a, the data of carbon isotope from refs.^14,21^).

The T-OAE was an oceanic anoxic event that occurred in the Toarcian period. The typical characteristic of the T-OAE is an apparent rapid negative organic carbon isotope excursion^19,21^. Our data show that the δ^13^C_org_ values of the Da’anzhai shale exhibit five obvious trends, and the data first show a negative trend (2950. 53–2958.21 m, trend 1 of δ^13^C_org_) and then a fluctuation from 2938.35 to 2949.78 m (trend 2 of δ^13^C_org_). The most noticeable negative carbon isotope excursion occurs between 2902.47 and 2936.05 m (trend 3 of δ^13^C_org_), and the δ^13^C_org_ value reaches − 29.8‰ (the negative carbon isotope excursion range is 3.4‰). Then, at 2899.07 m, the δ^13^C_org_ value changes to a positive excursion (trend 4 of δ^13^C_org_). In our study, the thickness of the most noticeable section of the negative carbon isotope excursion is approximately 36 m. The response of the T-OAE recorded in the Da’anzhai shale is characterized by a significant negative local excursion in the δ^13^C_org_ value^14,21^. In our research, the most noticeable section of negative carbon isotope excursion is located in the middle and upper parts of the middle submember and the bottom of the upper submember of the Da’anzhai member (Fig. 4). The lithology of this section is dark gray shale interbedded with limestone, and these petrographical features may indicate a deep paleowater depth. Moreover, the TOC content of the section with noticeable negative carbon isotope excursions remains high (Fig. 4, trend 2 of the TOC content). These phenomena correlate well with those of previous studies^14^. The δ^13^C_org_ values of the Da’anzhai member reported in previous studies are shown in Fig. 7. From west to east, three boreholes exhibit significant carbon isotope excursions. Among them, core A displays the most pronounced excursion, with an amplitude of approximately 4.3‰. The carbon isotope excursions in the LQ104X and LY1 wells are similar, with values of 3.8 and 3.4‰, respectively. The thicknesses of the carbon isotope excursion layers in cores A, LY1, and LQ104X are approximately 29.1, 33.6, and 30.8 m, respectively. According to previous discussions, the most notable negative carbon isotope excursions of the Da’anzhai shale in the study area (top at 2902.47 m and bottom at 2936. 05 m, trend 3 of δ^13^C_org_) can be identified as the section that shows the response to the T-OAE, and sections above and below the section that shows the response to the T-OAE are defined as unassociated T-OAE sections. Additionally, our data confirm the presence of T-OAE records in the central and northern Sichuan Basin, supporting the conclusion from a prior study that a basin-wide response to the T-OAE is recorded in the Sichuan Basin^14^.

### Paleosedimentary environmental reconstruction

#### OM origin

For lacustrine systems, primary productivity, redox conditions, and salinity are the essential parameters influencing OM accumulation^24^. In this section, different biomarkers are selected to reconstruct the paleosedimentary environment.

In this study, primary productivity in the water column is characterized by the type of OM; for example, the high contribution of aquatic organisms to kerogen may indicate high primary productivity. Rock–Eval pyrolysis effectively determines the kerogen type of OM^40^. Our results show that the OM of the Da’anzhai shale in the investigated area is type II_2_ and type III kerogen, indicating a mixed OM source of aquatic plankton and terrigenous higher plants. Moreover, organic petrographic observations indicate similar results: exinites are mainly sourced from aquatic algae, and a relatively high proportion of vitrinite in OM contributes to terrigenous higher plants. The distribution of the TI ratio is shown in Fig. 4, where the TI exhibits five apparent trends. Initially, the TI shows a decreasing trend, and in the section that shows the response to the T-OAE, it displays in increasing, a decreasing, and then an increasing trend (trend 2 to 4 of the TI). Furthermore, fluctuations in the δ^13^C_org_ value also signify changes in the source of OM. Generally, a negative excursion of the δ^13^C_org_ value is indicative of an increased proportion of aquatic organisms within the OM^41,42^. Consequently, the negative δ^13^C_org_ excursions observed in the section that shows the response to the T-OAE reinforce the findings from organic petrographic observations, supporting the interpretation of a significant shift in the origin of OM during this interval.

Molecular geochemical parameters, e.g., the n-alkane distribution, can also be used to assess the origins of OM^43,44^. C_27_ sterane is a marker of phytoplankton^45^, C_28_ is expected to be derived from phytoplankton containing chlorophyll^46^, and C_29_ sterane has various sources; in most cases, C_29_ sterane is derived from terrigenous higher plants^47^, while some researchers argue that C_29_ sterane may be derived from specific microalgae^48,49^. In this study, the ratio of steranes was used to characterize the OM source, e.g., a high (C_27_ + C_28_)/C_29_ sterane ratio indicates a high proportion of aquatic organisms accumulating OM. The (C_27_ + C_28_)/C_29_ ratio of the Da’anzhai member samples shows seven vertical trends, initially showing a downward trend (trend 1), followed by an upward–downward–upward and a relatively stable trend (trends 2–5). Above the T-OAE, the (C_27_ + C_28_)/C_29_ ratio exhibits a decreasing trend (trend 6). Combining pyrolysis tests, organic petrographical experiments, δ^13^C_org_ values, and biomarker data, the results indicate that OM was primarily sourced from the mixed contribution of aquatic algae and terrigenous higher plants. However, the sources of OM and primary productivity still exhibit certain differences. During the T-OAE period, aquatic organisms contributed more to OM, and the primary productivity was relatively high.

#### Redox conditions

Most of the samples indicate a dysoxic environment with Pr/Ph ratios greater than 1, while some samples with low Pr/Ph ratios indicate an intermittent reducing environment, demonstrating the impact of environmental fluctuations on lacustrine sedimentary systems (Table 4). The Pr/Ph ratio shows a decreasing trend in the section that shows the response to the T-OAE (Fig. 4, trend 2), followed by an obvious increasing trend above the T-OAE (trend 3). Our data indicate that the Da’anzhai shale experienced dysoxic to anoxic conditions during the T-OAE period, and the results reflect the impact of the T-OAE on the redox conditions of the Da’anzhai member.

#### 415Salinity and abnormal abundance of diahopanes

Salinity is another crucial factor affecting OM accumulation. Liu et al.^14^ used trace elements to evaluate the paloesalinity of the Da’anzhai member and concluded that the Da’anzhai member was primarily deposited in freshwater to brackish conditions. In most cases, the vertical stratification of the water column is caused by differences in salinity conditions. Therefore, the GI represents the water column salinity. A GI greater than 0.1 indicates water column stratification^50,51^. Our GI data show anomalously high values with a maximum of 19.11, indicating the high salinity of the water column. However, this result contradicts the results of previous studies involving inorganic geochemistry (Sr/Ba ratios ranging from 0.18 to 0.26, indicating freshwater to brackish salinity conditions)^14^ and molecular geochemistry (GI values ranging from 0.05 to 0.23)^15^. Subsequently, the samples showing anomalously high GI values exhibit low C_30_H and high C_30_D values (Table 5 shows high C_30_D/C_30_H values). Therefore, the abnormally high GI does not indicate water column stratification and salinity but rather a low C_30_H value. Earlier studies have reported several cases in which source rocks had abnormally high C_30_D/C_30_H ratios in the mature stage, such as the Permian Pebbley Beach Formation in the Sydney Basin^52^, the Triassic Yanchang Formation in the Ordos Basin^53^, and the Jurassic Xinhe Formation in the Yabulai Basin^54^. Researchers have proposed that abnormally high C_30_D/C_30_H values may be related to thermal maturity, the clay mineral content, salinity, and redox conditions^52–56^. At present, there is no accurate explanation for the abnormally high C_30_D/C_30_H ratios in rock extracts and oils^57^. From the data presented in Fig. 4, we can infer the reason for the abnormally high C_30_D/C_30_H ratios. First, the C_30_D/C_30_H values exhibit a similar trend to that of the clay mineral content, which may indicate that the catalysis of clay minerals plays an important role in the formation of these diahopanes. In the section that shows the response to the T-OAE, high clay mineral contents promoted the formation of diahopanes, which is consistent with findings from previous studies^15^. Additionally, the Pr/Ph ratio in this study indicates a suboxic environment during the deposition of the Da’anzhai member, which facilitated the formation of diahopanes^52,57^. It is important to note that Reolid et al.^15^ did not report abnormally high C_30_D/C_30_H values or abnormally low GI values in the study of the Da’anzhai member, likely due to the lower thermal maturity of their samples (Ro values of approximately 0.82–0.85%). C_30_D is more stable than C_30_H at high thermal maturity^58^, explaining the discrepancy in molecular geochemical characteristics between our study and that of Reolid et al.^15^. This discrepancy further underscores the significant heterogeneity in the molecular geochemical characteristics of lacustrine shales. Regardless of the generation mechanism of C_30_D, the abnormally high GI values are not derived from the high Gam content. Therefore, the GI is not a reliable indicator for assessing salinity conditions in this context. Our study emphasizes the need for caution when using the GI as a salinity proxy in environments characterized by clay mineral enrichment, high thermal maturity, and suboxic conditions. Furthermore, the concurrent findings of the increased clay mineral content in the section that shows the response to the T-OAE in the Da’anzhai member, as observed in both our study and that of Reolid et al.^15^, suggest that an abnormally high C_30_D/C_30_H ratio may serve as a potential proxy for pinpointing the section that shows the response to the T-OAE within this geological context.

Earlier studies have shown that an extended tricyclic terpane ratio (ETR = (C_28_TT + C_29_TT)/(C_28_TT + C_29_TT + Ts)) can characterize the salinity of lake water^59,60^. Table 5 shows the ETR data for the study area. The ETR values range from 0.27–0.71, indicating fluctuating salinity conditions. Combined with the Sr/Ba ratio of Liu et al.^14^, the salinity conditions in the Da’anzhai shale are mainly freshwater to brackish, and the ETR value can reflect the variation in salinity. The ETR data revealed four vertical trends (Fig. 4): decreasing values (trend 1) followed by consistently low values (trend 2). The later T-OAE exhibits increasing values (trend 3), after which the ETR values remain relatively high (trend 4).

#### Paleoenvironmental evolution of the Da’anzhai member

By integrating the above biomarker data, in this study, the sedimentary environment and evolutionary process of the Da’anzhai member in the study area are reconstructed. In the early Da’anzhai period, the lake was relatively small. The lake expanded from the lower to middle submembers, the sediment changed from coquina to black shale, the organic matter abundance increased (trend 1 of the TOC content), and the salinity of the water column decreased (trend 1 of the ETR). However, due to the increase in terrigenous influxes during this period (the clay mineral content increased, trend 1), the redox conditions did not shift to those of a reducing environment (trend 1 of Pr/Ph). This explanation is further supported by OM sources (trend 1 of the (C_27_ + C_28_)/C_29_ ratio and trend 1 of the TI). The middle submember of the Da’anzhai member was deposited mainly in a semideep lake to deep lake environment, and a significant negative excursion in the δ^13^C_org_ value (trend 3 of δ^13^C_org_) occurred after 2936.05 m, corresponding to the terrigenous response of the Da’anzhai member to the T-OAE. During this period, the temperature and precipitation increased (Yang, (2019)^61^ reported an increase in the C-values in the section that shows the response to the T-OAE in boreholes in the central Sichuan Basin), the lake further expanded, and the salinity remained relatively low (trend 2 of the ETR). The redox conditions changed to those of a relatively reducing environment (trend 2 of Pr/Ph), which is consistent with the results revealed by Mo_EF_ and Corg/P data^14^. The anoxic environment and high clay mineral content may also promote the formation of C_30_D, resulting in abnormally high GI values. In the section that shows the response to the T-OAE, the TOC content remained relatively high (trend 2 of the TOC content). Moreover, the increase in the proportion of aquatic organisms in OM (trend 2 of the (C_27_ + C_28_)/C_29_ ratio and trend 2 of TI) may represent an increase in primary productivity in the water column, which is consistent with the significant improvement in authigenic Cu and Ni contents during the T-OAE period reported by Yang^61^. Although there were obvious fluctuations in primary productivity during the T-OAE period, such as trend 3 for the (C_27_ + C_28_)/C_29_ ratio and trend 3 for the TI, the primary productivity generally remained relatively high in the section that shows the response to the T-OAE (trends 4 and 5 for the (C_27_ + C_28_)/C_29_ ratio and trend 3 for the TI). Notably, in the late T-OAE, the salinity increased (trend 3 of the ETR), which may indicate a decrease in the paleowater depth of the lake. Although the size of the lake decreased, the redox conditions did not significantly fluctuate (trend 2 of Pr/Ph), which may be attributed to the high primary productivity in the water column. The δ^13^C_org_ values showed a significant positive excursion (trend 3 of δ^13^C_org_), indicating the end of the T-OAE and significant changes in the sedimentary environment. At the end of the T-OAE, the primary productivity decreased (trend 6 of the (C_27_ + C_28_)/C_29_ ratio and trend 5 of the TI), leading to the anoxic environment changing to a dysoxic environment (trend 3 of the Pr/Ph), and a high salinity indicates a low paleowater depth (trend 4 of the ETR). The TOC content obviously decreased (trend 3 of the TOC content), and the sediments also changed from black shale to mudstone and limestone interbedded with sandstone.

### Mechanism of the response to the T-OAE and its effect on OM accumulation

Global events cause significant changes in sedimentary environments and lead to variations in TOC contents^25,62^. Hence, the mechanisms of OM enrichment and variations in the TOC content in the Da’anzhai member are discussed in this section. As mentioned above, the redox environment, primary productivity, and salinity conditions changed significantly during the T-OAE period. This leads to a difference in the TOC content. Due to the change in the paleoclimate, the paleowater depth of the lake increased, and a relatively reducing environment formed. Moreover, the prosperity of aquatic organisms has led to the improvement of primary productivity, which is further conducive to the formation of a relatively reducing environment. Although the paleowater depth of the lake may have declined in the late T-OAE, the anoxic environment could still have been maintained due to the high primary productivity of the water column. In summary, the OM enrichment in the section that shows the response to the T-OAE was caused by the combination of the paleoclimate, redox conditions and primary productivity.

The mechanism of the response of lacustrine systems to OAEs has been the subject of intense research. Two hypotheses for the mechanism of the response of the T-OAE recorded in the Da’anzhai member, Sichuan Basin, exist: transgression and alteration of hydrological conditions^14,21^. As mentioned previously, controversies about response mechanisms still exist. Our data show that there was no direct marine influence on the Da’anzhai shale and that the alteration of hydrological conditions changed the paleosedimentary environment. As reported by Xu et al.^21^, marine incursions occurred in the middle of the T-OAE period, which is obviously different from the conclusion drawn from the ETR data in this paper. Moreover, although the ETR tended to increase in the late T-OAE (trend 3), the salinity did not decrease significantly until the end of the T-OAE (trend 4 of the ETR). Furthermore, Wang et al.^33^ reconstructed the paleoenvironment based on elemental geochemistry from the same strata in the LY1 borehole. Their findings indicate that during the T-OAE period, the Sr/Ba ratios ranged from 0.15 to 0.28, with an average of 0.21. This ratio suggests only slight fluctuations in salinity within the water column, indicating conditions ranging from freshwater to brackish. Additionally, as depicted in Fig. 4, an increase in the clay mineral content also signifies a heightened influx of terrigenous debris. Together, these phenomena challenge the hypothesis of a transgressive event during this period. Therefore, the response of the Da’anzhai shale to alteration by the T-OAE was manifested in hydrological conditions rather than marine incursions. This study revealed the mechanism of the lacustrine response of the Da’anzhai shale to the T-OAE from the perspective of molecular organic geochemistry.

## Conclusions

The geological features of the Da’anzhai shales of the Lower Jurassic Ziliujing Formation were characterized. Three primary findings are listed as follows.

1. The TOC contents of the Da’anzhai shale are 0.10–3.63% (average of 1.61%). The T_max_ data and biomarkers indicate that the shales are in the mature to highly mature stage, and pyrolysis data and organic petrographical analysis show that type II_2_ and type III kerogens dominate in the Da’anzhai shale.
2. Generally, the Da’anzhai shales were deposited in a dysoxic transitional environment to an intermittent reducing environment with freshwater to brackish conditions. The terrigenous response to the T-OAE can be identified in the middle and upper parts of the middle submember and the bottom of the upper submember of the Da’anzhai member (with depths of 2902.47–2936.05 m). The sedimentary environment of the T-OAE period was anoxic, with higher primary productivity than that of the unassociated T-OAE section. It is important to exercise considerable caution when using the GI as a proxy for assessing salinity in clay mineral enrichment, high thermal maturity, and suboxic environments. The abnormally high C_30_D/C_30_H ratio may serve as a promising proxy for identifying the section that shows the response to the T-OAE in the Da’anzhai member.
3. The T-OAE changed the redox conditions, salinity, and primary productivity during the depositional period in the middle of the Da’anzhai member, which further resulted in OM enrichment. In the study area, the improvement in hydrological cycling rather than marine incursions is the response of the Da’anzhai shale to the T-OAE.

## Data Availability

Data sets generated during the current study are available from the corresponding author on reasonable request.
